# Blockade of Adenosine A_2A_ Receptor Protects Photoreceptors after Retinal Detachment by Inhibiting Inflammation and Oxidative Stress

**DOI:** 10.1155/2020/7649080

**Published:** 2020-07-02

**Authors:** Sha Gao, Na Li, Yanuo Wang, Yisheng Zhong, Xi Shen

**Affiliations:** ^1^The Department of Ophthalmology, Ruijin Hospital, Shanghai Jiao Tong University School of Medicine, 197, Ruijin 2nd Road, Shanghai 200025, China; ^2^The Department of Ophthalmology, Ruijin North Hospital, Shanghai Jiao Tong University School of Medicine, 999, Xiwang Road, Shanghai 201801, China

## Abstract

**Purpose:**

Adenosine A_2A_ receptor (A_2A_R) signaling is neuroprotective in some retinal damage models, but its role in neuronal survival during retinal detachment (RD) is unclear. We tested the hypothesis that A_2A_R antagonist ZM241385 would prevent photoreceptor apoptosis by inhibiting retinal inflammation and oxidative stress after RD.

**Methods:**

The A_2A_R antagonist ZM241385 was delivered daily to C57BL/6J mice for three days at a dose (3 mg/kg, i.p.) starting 2 hours prior to creating RD. A_2A_R expression, microglia proliferation and reactivity, glial fibrillary acidic protein (GFAP) accumulation, IL-1*β* expression, and reactive oxygen species (ROS) production were evaluated with immunofluorescence. Photoreceptor TUNEL was analyzed.

**Results:**

A_2A_R expression obviously increased and accumulated in microglia and Müller cells in the retinas after RD. The A_2A_R antagonist ZM241385 effectively inhibited retinal microglia proliferation and reactivity, decreased GFAP upregulation and proinflammatory cytokine IL-1*β* expression of Müller cells, and suppressed ROS overproduction, resulting in attenuation of photoreceptor apoptosis after RD.

**Conclusions:**

The A_2A_R antagonist ZM241385 is an effective suppressor of microglia proliferation and reactivity, gliosis, neuroinflammation, oxidative stress, and photoreceptor apoptosis in a mouse model of RD. This suggests that A_2A_R blockade may be an important therapeutic strategy to protect photoreceptors in RD and other CNS diseases that share a common etiology.

## 1. Introduction

Photoreceptor apoptosis because of physical separation of the photoreceptors from the retinal pigment epithelium (RPE) results in visual loss in a number of retinal diseases, including macular degeneration [1], retinopathy of prematurity [2], diabetic retinopathy [3], and retinal detachment (RD) [4, 5]. Photoreceptors are extremely vulnerable and undergo apoptosis in various types of RD including rhegmatogenous, tractional, and exudative. Although surgical treatment is routinely carried out to reattach the retina, visual acuity is not always restored because of RD-induced photoreceptor apoptosis [4, 5]. Currently, there are no effective therapies for protecting photoreceptors after RD. Therefore, the development of neuroprotective agents for photoreceptors is essential to provide visual stability for RD patients undergoing surgical treatment.

In RD, multiple pathways are known to be involved in the RD-induced photoreceptor apoptosis, including the caspase pathway, autophagy, inflammation and gliosis, and reactive oxygen species (ROS) [6–9]. Reactive microglia cells are prevalent in the detached retina where they play a principal role in RD-induced photoreceptor apoptosis [10]. Müller cells are central to the pathophysiology of RD, being involved in gliosis, initiation of inflammatory cascades, and proliferative responses [11]. Increased levels of proinflammatory mediator, IL-1*β*, are found in the retina and aqueous humor of RD patients and experimental RD [8, 12–14]. Excessive generation of ROS and the consequent induction of oxidative stress are one of the critical factors that trigger cellular response to RD [15] and are also a major cytotoxic factor for photoreceptor apoptosis [9, 16].

Adenosine is a neuromodulator in the central nervous system acting through the activation of four receptors, A_1_, A_2A_, A_2B_, and A_3_ [17]. The adenosine A_2A_ receptor (A_2A_R), a key molecule in the neural network, has been located on photoreceptors and RGCs of zebrafish [18], rat [19] and mouse [20], rat Müller cells [21], retinal pigment epithelium and choriocapillaris of rat [19], dog endothelium [22], and microglia of human [23] and rat [24]. A_2A_R participates in inducing and maintaining microglial reactivity [25], NO synthase-II expression [26], cyclooxygenase-2 (COX-2), and the synthesis and release of proinflammatory cytokines [26, 27] through the activation of its G-protein-coupled receptor [28]. Previous studies have demonstrated that pharmacological inhibition of A_2A_R affords profound neuroprotection in animal models of several cerebral diseases [29, 30]. Recent studies show that A_2A_R antagonists prevent photoreceptors and RGC apoptosis by modulating the inflammation and oxidative stress in both age-related macular degeneration (AMD) [31] and glaucoma [32]. However, whether A_2A_R-mediated neuroprotection is applicable to RD-induced photoreceptor apoptosis has not been determined.

In the current study, we have found that the expression of A_2A_R is obviously increased in microglia and Müller cells in the detached retina in a time-dependent manner, which is accompanied by enhanced microglia and Müller cell reactivity. Meanwhile, we also provided evidence that a selective A_2A_R antagonist, ZM241385, effectively protected photoreceptors with concomitant suppression of microglia activation, GFAP and proinflammatory cytokine IL-1*β* expression and ROS production after RD. Thus, in this study, we point out that A_2A_R is a potential therapeutic target for preventing RD-induced photoreceptor apoptosis.

## 2. Materials and Methods

### 2.1. Experimental Animals

We followed the methods of Su et al. [33]. All animal experiments followed the guidelines of the Association for Research in Vision and Ophthalmology Statement for the Use of Animals in Ophthalmic and Vision Research and were approved by Shanghai Jiao Tong University School of Medicine Animal Care and Use Committee. Male, 7–9 weeks old, C57BL/6J mice were allowed free access to water in a climate-controlled room with a 12 h light/12 h dark cycle.

### 2.2. Induction of RD

RD was induced as described previously [34]. Briefly, the mice were anesthetized with sodium pentobarbital (30 mg/kg, i.p.), and pupils were dilated with phenylephrine (5%) and tropicamide (0.5%). The temporal conjunctiva at the posterior limbus was incised. A 30-gauge needle (BD) was used to create a sclerotomy 1 mm posterior to the limbus. A corneal puncture was made with a 30-gauge needle to lower intraocular pressure. A 33-gauge needle connected to a Hamilton 10 *μ*L syringe was inserted into the subretinal cavity. Then, 4 *μ*L of 1% sodium hyaluronate (Provisc; Alcon) was injected, separating approximately 60% of the neurosensory retina from the underlying RPE. Eyes with subretinal hemorrhage or unsuccessful detachment were excluded from analysis. We detected retinas only within the area detached after RD by immunofluorescence staining.

### 2.3. Drug Administration

The mice were given the following treatment, as once daily i.p. injections of ZM241385 (S8105; Selleckchem, USA) at a dose of 3 mg/kg or vehicle for three days. The injected volumes did not exceed 0.2 mL per animal. The doses of the drugs were based on previous studies [35, 36].

### 2.4. Immunofluorescence of Retinal Sections

Immunofluorescence of retinal sections was performed as previously reported [33, 37]. The eyecups were cut into 10 *μ*m thick sections. The cryosections were, respectively, incubated with monoclonal mouse anti-mouse A_2A_R antibody (05-717; Millipore, USA), polyclonal rabbit anti-mouse Iba-1 antibody (019-19741; Wako, Japan), monoclonal rat anti-mouse CD11b antibody (ab8878; Abcam, USA), monoclonal rat anti-mouse MHC Class II antibody (ab25333; Abcam, USA), monoclonal mouse anti-mouse GFAP antibody (mab3402; Sigma-Aldrich, USA), polyclonal rabbit anti-mouse GFAP antibody (ab7260; Abcam, USA), and polyclonal rabbit anti-mouse IL-1*β* antibody (ab9722; Abcam, USA) by overnight incubation at 4°C. The cryosections were then, respectively, incubated in Alexa 555-conjugated anti-mouse IgG (4409), Alexa 488-conjugated anti-mouse IgG (4408), Alexa 555-conjugated anti-rabbit IgG (4413), Alexa 488-conjugated anti-rabbit IgG (4412), or Alexa 488-conjugated anti-rat IgG (4416) (all from cell signaling technology, Inc.). The sections were finally counterstained with DAPI (sc-3598; Santa Cruz Biotechnology, Inc.). Images were captured with a confocal microscope (SP5; Leica Microsystems, Inc., USA) with a fixed detection gain for each comparative section.

### 2.5. Oxidative Stress Assay

Superoxide dismutase (SOD) activity and malondialdehyde (MDA) were performed as previously reported [38]. Briefly, fresh retinal tissue was converted to 100 g/L of retina homogenates in a homogenizer filled. The homogenates were centrifuged at low temperature for 15 min at a speed of 3500 r/min. Proper amount of supernatant was given to perform tissue protein quantification. Levels of SOD and MDA were determined in accordance with the specifications of the SOD kit (Dojindo Molecular Technologies, Japan) and the MDA kit (Nanjing Jiancheng Bioengineering Institute, China). The protein concentration of the samples was determined using a BCA protein assay kit.

### 2.6. TUNEL Assay

The eyes were fixed in 4% paraformaldehyde overnight, then embedded in paraffin and sectioned at a thickness of 10 *μ*m. The TUNEL assay was performed using the *In Situ* Cell Death Detection Kit (11684795910; Roche Diagnostics GmbH, Germany) according to the manufacturer's instructions on the frozen sections as previously described. The number of TUNEL^+^ cells in the outer nuclear layer (ONL) was calculated in a masked fashion. The density of TUNEL^+^ cells in the ONL was counted using ImageJ 1.48v software. These measurements were carried out without knowledge of the treatment.

### 2.7. Western Blot Analysis

Retinas from experimental eyes were dissected from the RPE-choroid. The samples were homogenized and lysed in RIPA buffer. And protein concentrations were calculated by the BCA protein assay kit (P0009; Beyotime, China). The samples were resolved on 10% SDS-PAGE gels and transferred onto PVDF membranes (EMD Millipore Corporation, US). The membranes were incubated overnight at 4°C with primary antibodies against A_2A_R (05-717; Millipore, USA) and *β*-actin (CW0096S; CWBiotech, China). After being washed, the membranes were incubated with horseradish peroxidase-conjugated secondary antibodies (Jackson ImmunoResearch Inc., USA). The protein expression level was determined by densitometric analysis and normalized to the level of *β*-actin.

### 2.8. Quantitative RT-PCR Analysis

mRNA expression of A_2A_R was analyzed by qRT-PCR as previously reported [37]. Briefly, after total RNA was extracted from the retinas using the TRIzol Reagent (Invitrogen, Carlsbad, CA, USA); total RNA was used to synthesize complementary DNA (cDNA) with a reverse transcription kit (Takara, Japan). The reaction system was composed of specific primers, cDNA, and the SYBR Green qPCR Mix (Takara, Japan). Primers were synthesized by a private company (Sangon Biotech Co. Ltd., China). *β*-Actin served as the reference gene, and the expression of target genes was calculated as 2^-*△△*Ct^. Primer sequences used were designed as follows: murine A_2A_R forward, 5′-AGAGCAAGAGGCAGGTATCTC-3′ and reverse, 5′-CCCAAAGGCTTTCTCACGGA-3′;

### 2.9. Statistical Analysis

The data are presented as the mean ± standard deviation (mean ± SD). The differences among the groups were analyzed by Student's *t*-test or one-way ANOVA according to the normal distribution. All statistical analysis was performed using GraphPad software (Prism 8; GraphPad Software, Inc.). Values of *P* < 0.05 were considered statistically significant.

## 3. Results

### 3.1. A_2A_R Expression Is Increased after RD

**Figure 1:**
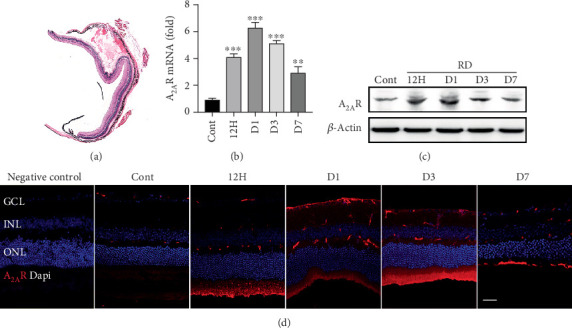
A_2A_R expression levels at different time after retinal detachment (RD) in mice. (a) Hematoxylin and eosin staining of retinal sections from mice after RD. (b) Quantification of A_2A_R mRNA in the retina of control and RD at 12 h, day 1, day 3, and day 7 (*n* = 6). (c) Western blot analysis of A_2A_R protein expression in the retina of control and RD mice at 12 h, day 1, day 3, and day 7 (*n* = 3). (d) Immunofluorescence staining of A_2A_R expression (red) in the retina of control and RD mice at 12 h, day 1, day 3, and day 7. ^∗∗^*P* < 0.01, ^∗∗∗^*P* < 0.005. Scale bar: 50 *μ*m.

To investigate the expression of A_2A_R in RD, we collected the mouse retinas from 12 h to day 7 after induction of RD (Figure 1(a)) and examined the mRNA and protein expression of A_2A_R using quantitative real-time PCR and Western blotting, respectively. Compared to the control, the mRNA expression of A_2A_R in retinas after RD increased. RT-PCR analysis revealed that there was a more than 6-fold induction of A_2A_R mRNA expression in the RD model at day 1 (Figure 1(b)). Meanwhile, immunoblots also showed high expression of A_2A_R protein in retinas after RD at 12 h, day 1, and day 3 (Figure 1(c)). Furthermore, to detect the distribution of A_2A_R in detached retina, the immunofluorescence staining was performed (Figure 1(d)). A_2A_R immunoreactivity was increased with time and very strong at day 1 in the detached retina, whereas there was minimal staining in the control. The A_2A_R staining was obviously detected in the ganglion cell layer (GCL), the inner plexiform layer (IPL), the inner nuclear layer (INL), and the outer plexiform layer (OPL) after RD. These results demonstrate a significant increase of A_2A_R mRNA and protein expression in the mouse retina after RD, specifically in the GCL, IPL, INL, and OPL.

### 3.2. A_2A_R Staining Is Colocalized with Microglia and Müller Cells after RD

**Figure 2:**
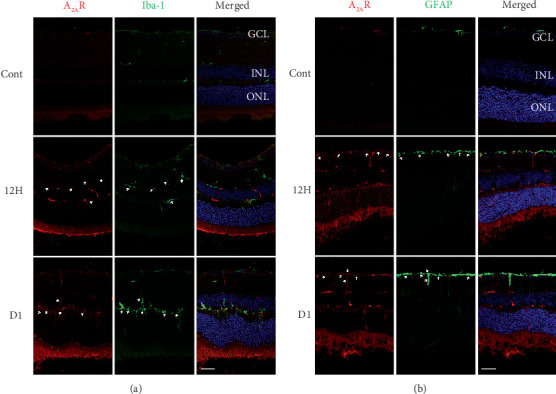
A_2A_R localization in microglia cells and Müller cells in the retina of the RD mouse model. (a) Retinal sections were stained with antibodies against A_2A_R (red) and Iba-1 (green) in the retina of control and RD mice at 12 h and day 1. The arrows designate region of colocalization of A_2A_R and microglia. (b) Retinal sections were stained with antibodies against A_2A_R (red) and GFAP (green) in the retina of control and RD mice at 12 h and day 1. The arrows designate region of colocalization of A_2A_R and Müller cells. Scale bar: 50 *μ*m.

To verify the identity of the A_2A_R^+^ cells, double immunofluorescence was performed from 12 h to day 1 after RD by using antibodies against A_2A_R and Iba-1, a marker of microglia cell (Figure 2(a)). Microglia was significantly increased in retina after RD, which is consistent with previous research. The A_2A_R and Iba-1 signals colocalized in microglia cells in the IPL, the inner nuclear layer (INL), the OPL, and the ONL (Figure 2(a), arrows). To verify the identity of the A_2A_R^+^ cells, double immunofluorescence was also performed after RD using antibodies against A_2A_R and GFAP, a Müller cell marker (Figure 2(b)). After RD, simultaneously upregulation of GFAP was detected with the increment of A_2A_R. Compared to the restricted expression of GFAP that was observed in the nerve fiber layer of the retina from the control eyes, the GFAP immunoreactivity was marked and extended across the entire neural retina to the ONL after RD. The A_2A_R and GFAP signals colocalized in Müller cells in the GCL and IPL (Figure 2(b), arrows). These data demonstrate that A_2A_R expression dramatically increased in the microglia and Müller cells in the retina after RD.

### 3.3. A_2A_R Blockade Inhibits Microglia Proliferation and Reactivity after RD

**Figure 3:**
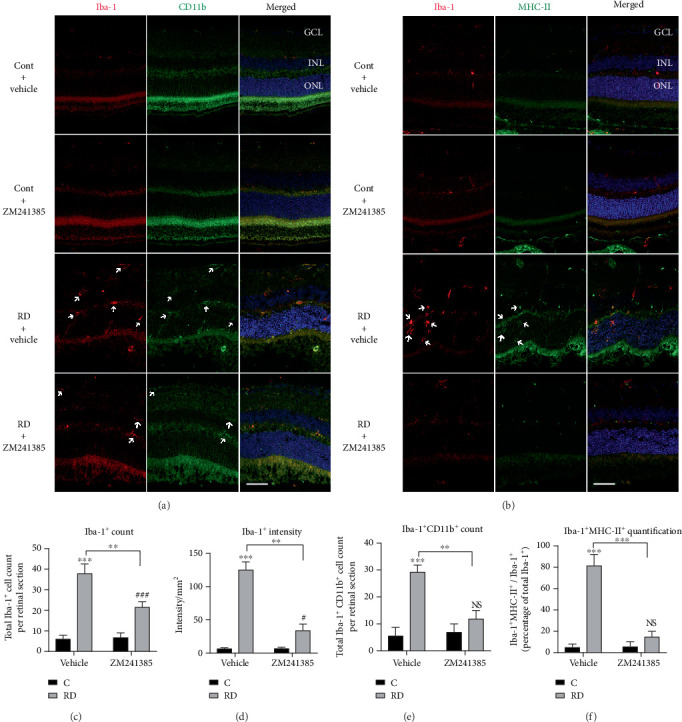
ZM241385 markedly reduced microglia proliferation and reactivity in the retina after RD in mice. (a) Retinal sections were stained with antibodies against Iba-1 (red) and CD11b (green). Representative images are depicted, and arrows indicate some Iba-1^+^ and CD11b^+^ cells found in each condition. (b) Retinal sections were stained with antibodies against Iba-1 (red) and MHC-II (green). Representative images are depicted, and arrows indicate some Iba-1^+^ and MHC-II^+^ cells found in each condition. (c) Quantification of Iba-1^+^ cell counts per retinal section in the retina (*n* = 8). (d) Quantification of Iba-1 intensity per mm^2^ in the retina (*n* = 8). (e) Quantification of Iba-1^+^ CD11b^+^ cell counts per retinal section in the retina (*n* = 8). (f) Activated microglia (Iba-1^+^ MHC-II^+^ cells) were counted and normalized to the percentage of total microglial cells (Iba-1^+^ cells) (*n* = 8). ^∗∗^*P* < 0.01, ^∗∗∗^*P* < 0.005, ^#^*P* < 0.05, ^###^*P* < 0.005, Scale bar: 50 *μ*m.

Microglia cells, the resident tissue macrophages of the retina, play a critical role in damage processes and have both neuroprotective and neurotoxic effects during retinal damage. To investigate the effects of A_2A_R on microglial response after RD, the immunofluorescence colocalization of Iba-1 (labeling both resting and active microglial cells) and CD11b (for active microglial cells staining) [39] was assessed in detached retina at day 3 (Figure 3(a)). Retinal Iba-1^+^ cell counts were significantly increased after RD, whereas Iba-1^+^ cell counts were strongly suppressed by ZM241385 compared with the control vehicle treatment after RD (Figure 3(c)). Significant accumulation of Iba-1 intensity was detected in detached retina compared to controls. However, the administration of ZM241385 obviously decreased Iba-1 intensity after RD (Figure 3(d)). Additionally, this blockade of microglial reactivity is illustrated by the colocalization of Iba-1 and CD11b. Iba-1^+^ CD11b^+^ cell counts were dramatically inhibited by ZM241385 after RD, compared with the control vehicle treatment (Figure 3(e)).

In order to better detect microglia reactivity, double labeling was also performed using primary antibodies to Iba-1 and major histocompatibility complex class II (MHC-II, highly expressed in reactive microglia) (Figure 3(b)). Iba-1^+^ MHC-II^+^ cells were considered reactive microglia. As expected, microglia reactivity was obviously increased in the retina after RD when compared with the retinas of control. ZM146385 administration showed a significant decrease in the percentage of reactive microglia cells compared to vehicle after RD, whereas the administration of ZM146385 to control animals did not change the number of reactive microglia (Figure 3(f)). These data suggest that A_2A_R is critical for the microglia proliferation and reactivity after RD and for the subsequent structural and functional disruption of these retinal layers.

### 3.4. A_2A_R Blockade Reduces Reactive Gliosis and Inflammatory Response after RD

**Figure 4:**
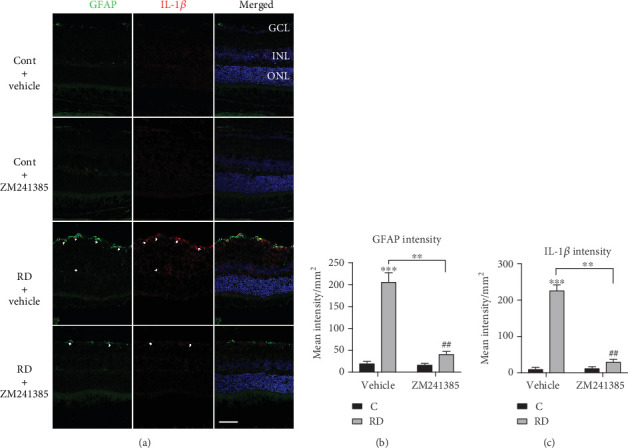
ZM241385 effectively inhibited upregulation of GFAP and IL-1*β* expression after RD in mice. (a) Retinal sections were stained with antibodies against GFAP (green) and IL-1*β* (red). The arrows designate the region of colocalization of GFAP and IL-1*β*. (b) Quantification of GFAP intensity per mm^2^ in the retina (*n* = 8). (c) Quantification of IL-1*β* intensity per mm^2^ in the retina (*n* = 8). ^∗∗^*P* < 0.01, ^∗∗∗^*P* < 0.005, ^##^*P* < 0.01. Scale bar: 50 *μ*m.

Activation of gliosis represents Müller glial remodeling in response to RD-induced retinal damage and contributes to tissue inflammation. Additionally, IL-1*β*, an important inflammatory cytokine, has previously been reported to contribute to the pathogenesis of photoreceptor apoptosis after RD [40]. Moreover, the blockade of A_2A_R has been shown to effectively prevent inflammatory responses within various injury models [41]. To investigate whether the A_2A_R blockade contributes to inhibiting reactive gliosis and inflammatory responses after RD, we evaluated the role of A_2A_R in the RD-induced GFAP and IL-1*β* expression. After RD, the level of GFAP was obviously upregulated, whereas the increased GFAP expression in detached retina was dramatically ameliorated by the addition of ZM241385 (Figures 4(a) and 4(b)). The expression of IL-1*β*, colocalized in Müller cells with GFAP signal, was detected in the GCL, the IPL, and the OPL at day 3 after RD (Figure 4(a)). The ZM146385 administration obviously suppressed the upregulation of IL-1*β* expression in Müller cells induced by RD (Figure 4(c)). Taken together, these results indicate that the A_2A_R blockade can inhibit RD-induced reactive gliosis and inflammatory response in Müller cells.

### 3.5. A_2A_R Blockade Suppresses the Oxidative Stress after RD

**Figure 5:**
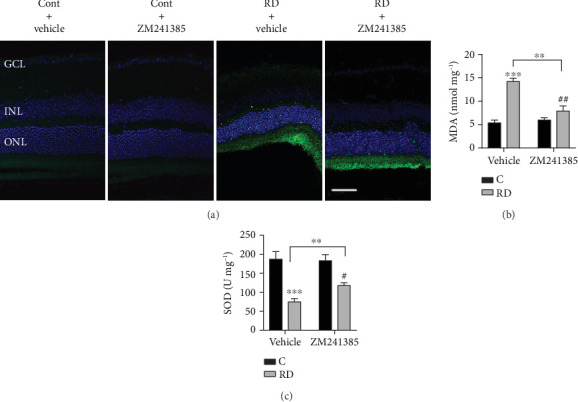
ZM241385 significantly inhibited oxidative stress in the retina after RD. (a) Immunofluorescence staining of ROS production (green) in retinal sections. (b) MDA concentration and (c) SOD activity analysis in the retina. ^∗∗^*P* < 0.01, ^∗∗∗^*P* < 0.005, ^#^*P* < 0.05, ^##^*P* < 0.01. Scale bar: 50 *μ*m.

Silencing of the caffeine-antagonized A_2A_R significantly reduced ROS production in THP-1 macrophages [42] and on UV-induced skin damage in mice [43]. We therefore hypothesized that A_2A_R blockade might protect neuronal cells from RD by reducing oxidative stress. To test the hypothesis, oxidative stress was measured in detached retina after ZM241385 administration. Immunofluorescence results showed that the upregulation of ROS in the ONL was inhibited dramatically by ZM241385 at day 3 after RD (Figure 5(a)). Moreover, we further observed that ZM241385 markedly restrained RD-induced MDA (Figure 5(b)), whereas the decreased activity of SOD was largely restored (Figure 5(c)). These results demonstrate that A_2A_R blockade effectively decreases RD-induced overproduction of oxidative stress.

### 3.6. A_2A_R Blockade Prevents Photoreceptor Apoptosis after RD

**Figure 6:**
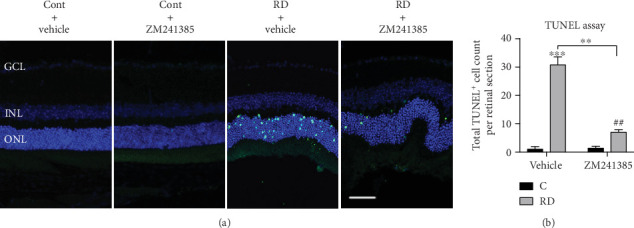
ZM241385 prevented RD-induced photoreceptor apoptosis in mice. (a) TUNEL labelling (green) in retinal sections after RD. (b) Quantification of TUNEL^+^ photoreceptors in the retina (*n* = 8). ^∗∗^*P* < 0.01, ^∗∗∗^*P* < 0.005, ^##^*P* < 0.01. Scale bar: 50 *μ*m.

Photoreceptor apoptosis was quantified at day 3 after RD which is the peak time point [44, 45]. To investigate whether A_2A_R is involved in RD-induced photoreceptor apoptosis, the TUNEL assay was performed among different groups (Figure 6(a)). In the absence of RD, the general appearance of the retina was similar at vehicle and ZM241385 groups. ZM241385 almost completely suppressed the appearance of TUNEL^+^ cells in the ONL after RD, whereas the control vehicle treatment had no effect (Figure 6(b)). These data show that A_2A_R plays a critical role in RD-induced photoreceptor apoptosis.

## 4. Discussion

Increased A_2A_R expression has been reported in several retinal disease models, including oxygen-induced retinopathy [22], diabetic retinopathy [46], glaucoma [41], and light-induced retinal degeneration [47]. Therefore, A_2A_R may be a critical factor for the inflammatory response during various acute and chronic retinal diseases. In the light-induced retinal degeneration model, A_2A_R upregulation is detected in the GCL and INL, coinciding with the massive apoptosis of photoreceptors [47]. In the current study, A_2A_R protein was detected in the GCL, IPL, INL, and OPL in the detached retina and expressed predominately in microglia and Müller cells after RD. In our conditions, RD-induced upregulation of A_2A_R in microglia and Müller cells suggested that microglia and Müller cells reacted to changes in retinal ischemia and A_2A_R modulated the response of microglia and Müller cells to retinal ischemia. The colocalization of A_2A_R^+^ microglia cells and the increased number of infiltrated microglia in mice after RD indicated that increased A_2A_R in microglia cells may attract microglia toward the outer retina. Additionally, the GFAP immunoreactivity in A_2A_R^+^ Müller cells was marked and extended across the entire neural retina to the ONL after RD. These findings are consistent with the fact that the outer retina is the main site of injury after RD and reveal that A_2A_R plays an important role in the photoreceptor cell death in detached retina.

There is a controversy on the effects mediated by A_2A_R in pathological conditions, since A_2A_R activation in peripheral immune cells is anti-inflammatory [48, 49], and in chronic conditions of the central nervous system, the blockade of the A_2A_R confers protection [28]. Recently, A_2A_R blockade has been shown to selectively reduce avascular areas and neovascularization, with the decreased cellular apoptosis and proliferation, and increased astrocyte and tip cell functions in OIR [50]. And the administration of A_2A_R blockade, ZM241385, has been illustrated to reduce microglia activation and decrease the proinflammatory factor expression to improve RGC survival in experimental glaucoma [41]. Moreover, Boia et al. [24] have demonstrated that treatment with A_2A_R antagonist KW6002 and caffeine intake could obviously inhibit microglia reactivity and effectively protect retina against transient ischemic damage. A_2A_R selective antagonist SCH58261 has been revealed to decrease GFAP expression in rat brain astrocyte cell line with ischemia-like injury [51]. However, whether A_2A_R blockade could play a neuronal protective role in RD is still unclear. In the present study, we observed that ZM241385, a selective A_2A_R antagonist, inhibited microglia reactivity after RD, accompanied by reduced proliferation of microglia. We found that ZM241385 also decreased GFAP expression and alleviated expression of inflammatory cytokine IL-1*β*. Furthermore, our results revealed that ZM241385 reduced obviously ROS production and attenuated the increase in MDA concentration after RD, while SOD activity increased in detached retinas. This was evident in a reduction of oxidative stress induced by RD, through administration of A_2A_R blockade.

Mounting evidence indicates that the microglia activation may contribute to the secondary injury to neurons and result in the chronic neuroinflammation [52]. The activation of microglia leads to the excessive releasement of proinflammatory cytokines, such as IL-1*β*, IL-6, and TNF-*α* [53]. Although the release of these proinflammatory cytokines is intended to protect the central nervous system tissue from further damage, they can impair simultaneously neurons [54]. Interestingly, the blockade of A_2A_R affords neuroprotection in several models of neurodegeneration, including in the retina [28, 41, 55–57]. One of the mechanisms that explains the protective properties of A_2A_R antagonists is the control of microglia-mediated neuroinflammation [28, 56]. It has also been illustrated that A_2A_R blockade confers neuroprotection by controlling microglia reactivity in vivo [31] and in glaucoma [58]. Likewise, we found that ZM241385 could dramatically inhibit the increase of Iba-1 intensity and count of activated microglial cells after RD in the current study. Our finding that A_2A_R blockade could alter microglia reactivity is consistent with those in other experimental models of retinal damage [32].

GFAP is a marker of gliosis and is increased in reactive Müller glia cells in various retinal disorders. Increasing studies have reported that GFAP levels in the central nervous system are elevated under pathological conditions and that this process plays an important role in neural injury [59, 60]. However, the pathological mechanisms of Müller activation and GFAP upregulation after RD are largely unknown. As a consequence of RD, a “mechanical” damage to the retina, GFAP expression increases obviously in Müller cells. In addition, the hypertrophy of Müller both within the retina and on the photoreceptor is accompanied with GFAP upregulation [61]. Previous studies have shown that the deletion of GFAP can prevent RD-induced gliosis and rescue photoreceptor degeneration, which highlights the key role of Müller cells in regulating retinal damage [62]. In this study, we found that ZM241385 effectively suppressed the upregulation of GFAP expression in Müller cells. The results suggest that A_2A_R blockade could prevent Müller activation after RD. However, improvement is needed in order to assess the effect of A_2A_R regulation in a therapeutic setting using a modified model of A_2A_R antagonist administration after the induction of RD.

IL-1*β* is a critical inflammatory cytokine involved in various retinal diseases [58, 63–65]. Previous studies have suggested IL-1*β* plays an important role in inducing photoreceptor apoptosis, using models of retinopathy of prematurity [66] and age-related macular degeneration [40, 67]. Kataoka et al. [40] showed that IL-1*β* partially contributed to photoreceptor cell apoptosis after RD utilizing caspase-1 inhibitor or IL-1*β* neutralizing antibody, which is in contrast to previous studies that have shown that IL-1*β* administration into the subretinal space does not increase photoreceptor apoptosis during RD [68]. Interestingly, Zhao and colleagues reported that caffeine could inhibit the increased production of IL-1*β* by suppressing A_2A_R signaling to prevent LPS-induced THP-1 macrophage activation [42]. In this study, we found that IL-1*β* expression was significantly increased and located in the Müller cells after RD as it was previously observed [40, 68], whereas the upregulation of IL-1*β* dramatically decreased after ZM241385 administration. The results suggest that A_2A_R blockade could effectively prevent RD-induced upregulation of IL-1*β* expression in Müller cells.

The cellular and molecular mechanisms inducing photoreceptor cell death have been partially revealed. Recent studies have shown that RIP kinase-mediated necrotic signaling [69] and FAS-mediated apoptosis pathway [70] contribute to photoreceptor death after RD. However, accumulating evidence suggests that the rapid increase of oxidative stress is currently considered to be a critical event for irreversible cellular damage in RD [14]. Previous studies have demonstrated an increased generation of ROS after RD, whereas photoreceptor cell death can be prevented after RD by suppressing ROS [71, 72]. The overproduction of ROS is known to interact with various inflammatory cytokines, including IL-1*β*, TNF-*α*, and CCL2, which suggests an important role for ROS in mediating the stress response [73]. Concurrent with this, previous studies have demonstrated that microglia A_2A_R blockade suppresses elevated pressure-induced oxidative stress in retina [32]. Furthermore, modulation of *α*-adrenoceptor signaling protects photoreceptors from apoptosis after RD by inhibiting ROS production [14]. In our work, we showed that ZM241385 was able to prevent photoreceptor loss from ROS overproduction triggered after RD, further reinforcing its role in controlling retinal neuroinflammation. We first found that MDA concentration increased in the detached retina, while the SOD activity decreased significantly after RD. Secondly, we observed that accompanied by a decrease in MDA and an increase in SOD, ZM241385 inhibited photoreceptor apoptosis after RD. This suggests that oxidative stress plays a key role in nerve injury after RD. Therefore, pharmacological therapies targeting oxidative stress may be critical for inhibiting RD-induced photoreceptor apoptosis.

## 5. Conclusions

In conclusion, we demonstrate that A_2A_R expression significantly upregulate and its colocalization with microglia and Müller cells in the retina after RD. In addition, A_2A_R blockade could provide effective protection against photoreceptor apoptosis in a mouse model of experimental RD for the first time. Meanwhile, we further demonstrate that the neuroprotective effects of the A_2A_R antagonist ZM241385 is related to the amelioration of RD-induced environmental stress that leads to microglia proliferation and reactivity, reactive gliosis, upregulation of proinflammatory cytokine, and activation of oxidative stress. Our results suggest that A_2A_R blockade may present novel therapeutic targets for strategies aimed at preserving visual acuity in patients with RD.

## Data Availability

The data used to support the findings of this study are available from the corresponding author upon request.

## References

[1] Dunaief J. L., Dentchev T., Ying G.-S., Milam A. H. (2002). The role of apoptosis in age-related macular degeneration. *Archives of Ophthalmology*.

[2] Fulton A. B., Hansen R. M., Petersen R. A., Vanderveen D. K. (2001). The rod photoreceptors in retinopathy of prematurity: an electroretinographic study. *Archives of Ophthalmology*.

[3] Barber A. J., Lieth E., Khin S. A., Antonetti D. A., Buchanan A. G., Gardner T. W. (1998). Neural apoptosis in the retina during experimental and human diabetes. Early onset and effect of insulin. *The Journal of Clinical Investigation*.

[4] Cook B., Lewis G. P., Fisher S. K., Adler R. (1995). Apoptotic photoreceptor degeneration in experimental retinal detachment. *Investigative Ophthalmology & Visual Science*.

[5] Arroyo J. G., Yang L., Bula D., Chen D. F. (2005). Photoreceptor apoptosis in human retinal detachment. *American Journal of Ophthalmology*.

[6] Kiang L., Ross B. X., Yao J. (2018). Vitreous cytokine expression and a murine model suggest a key role of microglia in the inflammatory response to retinal detachment. *Investigative Ophthalmology & Visual Science*.

[7] Matsumoto H., Sugio S., Seghers F. (2018). Retinal detachment-induced Müller glial cell swelling activates TRPV4 ion channels and triggers photoreceptor death at body temperature. *The Journal of Neuroscience*.

[8] Nakazawa T., Hisatomi T., Nakazawa C. (2007). Monocyte chemoattractant protein 1 mediates retinal detachment-induced photoreceptor apoptosis. *Proceedings of the National Academy of Sciences of the United States of America*.

[9] Carmody R. J., McGowan A. J., Cotter T. G. (1999). Reactive oxygen species as mediators of photoreceptor apoptosis *in vitro*. *Experimental Cell Research*.

[10] Fischer A. J., Zelinka C., Milani-Nejad N. (2015). Reactive retinal microglia, neuronal survival, and the formation of retinal folds and detachments. *Glia*.

[11] Bringmann A., Pannicke T., Grosche J. (2006). Muller cells in the healthy and diseased retina. *Progress in Retinal and Eye Research*.

[12] Dai Y., Wu Z., Sheng H., Zhang Z., Yu M., Zhang Q. (2015). Identification of inflammatory mediators in patients with rhegmatogenous retinal detachment associated with choroidal detachment. *Molecular Vision*.

[13] Xie J., Zhu R., Peng Y. (2017). Tumor necrosis factor-alpha regulates photoreceptor cell autophagy after retinal detachment. *Scientific Reports*.

[14] Li T., Yang S., She X. (2019). Modulation of *α*-adrenoceptor signalling protects photoreceptors after retinal detachment by inhibiting oxidative stress and inflammation. *British Journal of Pharmacology*.

[15] Zacks D. N., Han Y., Zeng Y., Swaroop A. (2006). Activation of signaling pathways and stress-response genes in an experimental model of retinal detachment. *Investigative Ophthalmology & Visual Science*.

[16] Rotstein N. P., Politi L. E., German O. L., Girotti R. (2003). Protective effect of docosahexaenoic acid on oxidative stress-induced apoptosis of retina photoreceptors. *Investigative Ophthalmology & Visual Science*.

[17] Ciruela F. (2011). Adenosine receptors. *Biochimica et Biophysica Acta*.

[18] Grillo S. L., McDevitt D. S., Voas M. G., Khan A. S., Grillo M. A., Stella S. L. (2019). Adenosine receptor expression in the adult zebrafish retina. *Purinergic Signal*.

[19] Kvanta A., Seregard S., Sejersen S., Kull B., Fredholm B. B. (1997). Localization of adenosine receptor messenger RNAs in the rat eye. *Experimental Eye Research*.

[20] Li H., Zhang Z., Blackburn M. R., Wang S. W., Ribelayga C. P., O’Brien J. (2013). Adenosine and dopamine receptors coregulate photoreceptor coupling via gap junction phosphorylation in mouse retina. *The Journal of Neuroscience*.

[21] Newman E. A. (2005). Calcium increases in retinal glial cells evoked by light-induced neuronal activity. *The Journal of Neuroscience*.

[22] Taomoto M., McLeod D., Merges C., Lutty G. A. (2000). Localization of adenosine A2a receptor in retinal development and oxygen-induced retinopathy. *Investigative Ophthalmology & Visual Science*.

[23] Angulo E., Casadó V., Mallol J. (2003). A1 adenosine receptors accumulate in neurodegenerative structures in Alzheimer disease and mediate both amyloid precursor protein processing and tau phosphorylation and translocation. *Brain Pathology*.

[24] Boia R., Elvas F., Madeira M. H. (2017). Treatment with A_2A_ receptor antagonist KW6002 and caffeine intake regulate microglia reactivity and protect retina against transient ischemic damage. *Cell Death & Disease*.

[25] Haskó G., Linden J., Cronstein B., Pacher P. (2008). Adenosine receptors: therapeutic aspects for inflammatory and immune diseases. *Nature Reviews. Drug Discovery*.

[26] Saura J., Angulo E., Ejarque A. (2005). Adenosine A2A receptor stimulation potentiates nitric oxide release by activated microglia. *Journal of Neurochemistry*.

[27] Minghetti L., Greco A., Potenza R. L. (2007). Effects of the adenosine A2A receptor antagonist SCH 58621 on cyclooxygenase-2 expression, glial activation, and brain-derived neurotrophic factor availability in a rat model of striatal neurodegeneration. *Journal of Neuropathology and Experimental Neurology*.

[28] Gomes C. V., Kaster M. P., Tomé A. R., Agostinho P. M., Cunha R. A. (2011). Adenosine receptors and brain diseases: neuroprotection and neurodegeneration. *Biochimica et Biophysica Acta*.

[29] Atack J. R., Shook B. C., Rassnick S. (2014). JNJ-40255293, a novel adenosine A2A/A1Antagonist with efficacy in preclinical models of Parkinson's disease. *ACS Chemical Neuroscience*.

[30] Kanda T., Uchida S. (2014). Clinical/pharmacological aspect of adenosine A_2A_ receptor antagonist for dyskinesia. *International Review of Neurobiology*.

[31] Madeira M. H., Rashid K., Ambrósio A. F., Santiago A. R., Langmann T. (2018). Blockade of microglial adenosine A2A receptor impacts inflammatory mechanisms, reduces ARPE-19 cell dysfunction and prevents photoreceptor loss *in vitro*. *Scientific Reports*.

[32] Aires I. D., Boia R., Rodrigues‐Neves A. C. (2018). Blockade of microglial adenosine A2Areceptor suppresses elevated pressure-induced inflammation, oxidative stress, and cell death in retinal cells. *Glia*.

[33] Su T., Zhong Y., Demetriades A. M. (2018). Endocan blockade suppresses experimental ocular neovascularization in mice. *Investigative Ophthalmology & Visual Science*.

[34] Matsumoto H., Miller J. W., Vavvas D. G. (2013). Retinal detachment model in rodents by subretinal injection of sodium hyaluronate. *Journal of Visualized Experiments*.

[35] Caiazzo E., Maione F., Morello S. (2016). Adenosine signalling mediates the anti-inflammatory effects of the COX-2 inhibitor nimesulide. *Biochemical Pharmacology*.

[36] Zygmunt M., Gołembiowska K., Drabczyńska A., Kieć-Kononowicz K., Sapa J. (2015). Anti-inflammatory, antioxidant, and antiparkinsonian effects of adenosine A_2A_ receptor antagonists. *Pharmacology, Biochemistry, and Behavior*.

[37] Gao S., Li C., Zhu Y. (2017). PEDF mediates pathological neovascularization by regulating macrophage recruitment and polarization in the mouse model of oxygen-induced retinopathy. *Scientific Reports*.

[38] Xie L., Yu S., Yang K., Li C., Liang Y. (2017). Hydrogen sulfide inhibits autophagic neuronal cell death by reducing oxidative stress in spinal cord ischemia reperfusion injury. *Oxidative Medicine and Cellular Longevity*.

[39] Tadmouri A., Champagnat J., Morin-Surun M. P. (2014). Activation of microglia and astrocytes in the nucleus tractus solitarius during ventilatory acclimatization to 10% hypoxia in unanesthetized mice. *Journal of Neuroscience Research*.

[40] Kataoka K., Matsumoto H., Kaneko H. (2015). Macrophage- and RIP3-dependent inflammasome activation exacerbates retinal detachment-induced photoreceptor cell death. *Cell Death & Disease*.

[41] Liu X., Huang P., Wang J. (2016). The effect of A2A receptor antagonist on microglial activation in experimental glaucoma. *Investigative Ophthalmology & Visual Science*.

[42] Zhao W., Ma L., Cai C., Gong X. (2019). Caffeine inhibits NLRP3 inflammasome activation by suppressing MAPK/NF-*κ*B and A2aR signaling in LPS-induced THP-1 macrophages. *International Journal of Biological Sciences*.

[43] Li Y. F., Ouyang S. H., Tu L. F. (2018). Caffeine protects skin from oxidative stress-induced senescence through the activation of autophagy. *Theranostics*.

[44] Hisatomi T., Sakamoto T., Goto Y. (2009). Critical role of photoreceptor apoptosis in functional damage after retinal detachment. *Current Eye Research*.

[45] Yang L., Bula D., Arroyo J. G., Chen D. F. (2004). Preventing retinal detachment-associated photoreceptor cell loss in Bax-deficient mice. *Investigative Ophthalmology & Visual Science*.

[46] Aires I. D., Madeira M. H., Boia R. (2019). Intravitreal injection of adenosine A_2A_ receptor antagonist reduces neuroinflammation, vascular leakage and cell death in the retina of diabetic mice. *Scientific Reports*.

[47] Soliño M., Larrayoz I. M., López E. M. (2018). The expression of adenosine receptors changes throughout light induced retinal degeneration in the rat. *Neuroscience Letters*.

[48] Hasko G., Pacher P. (2008). A2A receptors in inflammation and injury: lessons learned from transgenic animals. *Journal of Leukocyte Biology*.

[49] Sitkovsky M. V., Ohta A. (2005). The 'danger' sensors that STOP the immune response: the A2 adenosine receptors?. *Trends in Immunology*.

[50] Zhou R., Zhang S., Gu X. (2018). Adenosine A2A receptor antagonists act at the hyperoxic phase to confer protection against retinopathy. *Molecular Medicine*.

[51] Ke R. H., Xiong J., Liu Y., Ye Z. R. (2009). Adenosine A2a receptor induced gliosis via Akt/NF-*κ*B pathway in vitro. *Neuroscience Research*.

[52] Lee Y., Lee S.-R., Choi S. S., Yeo H.-G., Chang K.-T., Lee H. J. (2014). Therapeutically targeting neuroinflammation and microglia after acute ischemic stroke. *BioMed Research International*.

[53] Ellis-Behnke R. G., Jonas R. A., Jonas J. B. (2013). The microglial system in the eye and brain in response to stimuli in vivo. *Journal of Glaucoma*.

[54] Lull M. E., Block M. L. (2010). Microglial activation and chronic neurodegeneration. *Neurotherapeutics*.

[55] Boia R., Ambrosio A. F., Santiago A. R. (2016). Therapeutic opportunities for caffeine and A2A receptor antagonists in retinal diseases. *Ophthalmic Research*.

[56] Cunha R. A. (2005). Neuroprotection by adenosine in the brain: from a (1) receptor activation to a (2A) receptor blockade. *Purinergic Signal*.

[57] Madeira M. H., Boia R., Santos P. F., Ambrósio A. F., Santiago A. R. (2015). Contribution of microglia-mediated neuroinflammation to retinal degenerative diseases. *Mediators of Inflammation*.

[58] Madeira M. H., Elvas F., Boia R. (2015). Adenosine A2AR blockade prevents neuroinflammation-induced death of retinal ganglion cells caused by elevated pressure. *Journal of Neuroinflammation*.

[59] Pekny M., Pekna M. (2004). Astrocyte intermediate filaments in CNS pathologies and regeneration. *The Journal of Pathology*.

[60] Pekny M., Wilhelmsson U., Pekna M. (2014). The dual role of astrocyte activation and reactive gliosis. *Neuroscience Letters*.

[61] Lewis G. P., Fisher S. K. (2003). Up-regulation of glial fibrillary acidic protein in response to retinal injury: its potential role in glial remodeling and a comparison to vimentin expression. *International Review of Cytology*.

[62] Nakazawa T., Takeda M., Lewis G. P. (2007). Attenuated glial reactions and photoreceptor degeneration after retinal detachment in mice deficient in glial fibrillary acidic protein and vimentin. *Investigative Ophthalmology & Visual Science*.

[63] Tezel G., Wax M. B. (2000). Increased production of tumor necrosis factor-alpha by glial cells exposed to simulated ischemia or elevated hydrostatic pressure induces apoptosis in cocultured retinal ganglion cells. *The Journal of Neuroscience*.

[64] Wang J. W., Chen S. D., Zhang X. L., Jonas J. B. (2016). Retinal microglia in glaucoma. *Journal of Glaucoma*.

[65] Yuan L., Neufeld A. H. (2001). Activated microglia in the human glaucomatous optic nerve head. *Journal of Neuroscience Research*.

[66] Zhou T. E., Rivera J. C., Bhosle V. K. (2016). Choroidal involution is associated with a progressive degeneration of the outer retinal function in a model of retinopathy of prematurity: early role for IL-1*β*. *The American Journal of Pathology*.

[67] Hu S. J., Calippe B., Lavalette S. (2015). Upregulation of P2RX7 in Cx3cr1-deficient mononuclear phagocytes leads to increased Interleukin-1 secretion and photoreceptor neurodegeneration. *The Journal of Neuroscience*.

[68] Nakazawa T., Matsubara A., Noda K. (2006). Characterization of cytokine responses to retinal detachment in rats. *Molecular Vision*.

[69] Murakami Y., Miller J. W., Vavvas D. G. (2011). RIP kinase-mediated necrosis as an alternative mechanisms of photoreceptor death. *Oncotarget*.

[70] Zacks D. N., Zheng Q. D., Han Y., Bakhru R., Miller J. W. (2004). FAS-mediated apoptosis and its relation to intrinsic pathway activation in an experimental model of retinal detachment. *Investigative Ophthalmology & Visual Science*.

[71] Trichonas G., Murakami Y., Thanos A. (2010). Receptor interacting protein kinases mediate retinal detachment-induced photoreceptor necrosis and compensate for inhibition of apoptosis. *Proceedings of the National Academy of Sciences of the United States of America*.

[72] Roh M. I., Murakami Y., Thanos A., Vavvas D. G., Miller J. W. (2011). Edaravone, an ROS scavenger, ameliorates photoreceptor cell death after experimental retinal detachment. *Investigative Ophthalmology & Visual Science*.

[73] Blaser H., Dostert C., Mak T. W., Brenner D. (2016). TNF and ROS crosstalk in inflammation. *Trends in Cell Biology*.

